# Regulations of Tumor Microenvironment by Prostaglandins

**DOI:** 10.3390/cancers15123090

**Published:** 2023-06-07

**Authors:** Jeffrey Z. Nie, Man-Tzu Wang, Daotai Nie

**Affiliations:** 1 Department of Medical Microbiology, Immunology and Cell Biology, School of Medicine, Simmons Cancer Institute, Southern Illinois University, Springfield, IL 62702, USA; 2 Hillman Cancer Center, University of Pittsburg School of Medicine, Pittsburg, PA 15232, USA

**Keywords:** prostaglandins, tumor microenvironment, cyclooxygenase, non-steroid anti-inflammatory drugs, immunotherapy, eicosanoids, PGE_2_, PD1, PD-L1

## Abstract

**Simple Summary:**

Tumor evasion of immune surveillance is a hallmark of cancer posing challenges to effective cancer treatment. Herein we review the contributions of prostaglandins in shaping tumor microenvironments to modulate immune responses and cancer progression. Opportunities to target prostaglandins and their signaling receptors in improving cancer therapy, particularly immunotherapy, are explored.

**Abstract:**

Prostaglandins, the bioactive lipids generated from the metabolism of arachidonic acid through cyclooxygenases, have potent effects on many constituents of tumor microenvironments. In this review, we will describe the formation and activities of prostaglandins in the context of the tumor microenvironment. We will discuss the regulation of cancer-associated fibroblasts and immune constituents by prostaglandins and their roles in immune escapes during tumor progression. The review concludes with future perspectives on improving the efficacy of immunotherapy through repurposing non-steroid anti-inflammatory drugs and other prostaglandin modulators.

## 1. Prostaglandins: An Overview

Prostaglandins are bioactive eicosanoids involved in a number of homeostatic biological functions and inflammation [1]. They are formed through the metabolism of arachidonic acid through cyclooxygenase (COX), followed by different isomerases. Under normal conditions, this ω-6-unsaturated 20-carbon fatty acid is covalently linked to the *sn*-2 position of glycerophospholipids as a component of cellular membranes. Its release is under tight metabolic and physiologic regulation. During cellular responses to a number of cytokines, growth factors, or other hormones, arachidonic acid can be released from the plasma membrane through secretory, cytoplasmic, or both types of phospholipase A_2_ (sPLA_2_, cPLA_2_) [2], and then subsequently converted to various bioactive lipids, termed eicosanoids. These eicosanoids can function as the second messenger or through their cognate receptors, evoking various cellular responses.

**Figure 1:**
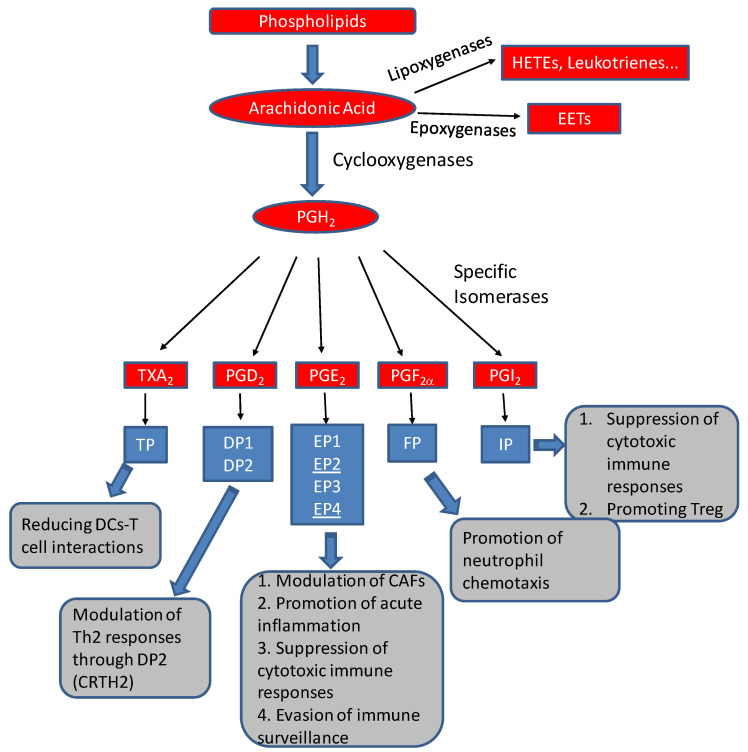
Formation of prostanoids and other eicosanoids (red boxes) by arachidonic acid metabolism, their cognate receptors (blue boxes), and their potential activities in tumor microenvironments (gray boxes).For example, PGE_2_, together with its two receptors, EP2/EP4 (underlined), can modulate cancer-associated fibroblasts (CAFs) [11], suppress cytotoxic immune responses [12,13], and help tumors to evade immune surveillance [14].

Arachidonic acid can be utilized by cyclooxygenase (COX), lipoxygenase (LOX), or P-450 epoxygenase pathways to form various eicosanoids (Figure 1). The COX pathway of arachidonic acid metabolism can form five primary prostanoids: prostaglandin D_2_, prostaglandin E_2_, prostaglandin F_2α_, prostaglandin I_2_, and thromboxane A_2_. The formation of prostanoids requires the formation of prostaglandin endoperoxide H_2_ (PGH_2_) through oxygenation by COX (also known as prostaglandin H_2_ synthases, PGHS); and the subsequent conversion of PGH_2_ to five primary prostanoids via specific synthases (or isomerases) [3,4,5,6,7,8]. All five primary prostanoids, PGD_2_, PGE_2_, PGF_2_, PGI_2_ (prostacyclin), and TxA_2_ (thromboxane A_2_), have potent biological activities, regulating immune functions such as gastric mucosa protection, kidney development and homeostasis, reproductive biology including embryo implantation, labor and uterine functions, and gastrointestinal integrity. They, particularly TXA2, can also modulate platelet aggregation, the sleep–wake cycle, and body temperature regulation [9]. There are two COX isoforms, with COX1 constitutively expressed in most cells and COX2 expression stimulated by various stimuli. Deregulated expression of COX2 has been extensively documented in various cancers. In one of our published studies, thromboxane synthase is frequently upregulated in prostate cancer, with its arachidonate product, thromboxane A_2_, involved in regulating tumor cytoskeleton reorganization and cell motility [10].

Besides the COX pathway, bioactive eicosanoids can be generated through lipoxygenase and epoxygenase pathways. Lipoxygenases are a family of non-heme iron-containing enzymes that oxygenate polyunsaturated fatty acids containing the 1-*cis*-4-*cis*-pentadiene moiety to form bioactive lipids. Metabolism of arachidonic acid by lipoxygenases can form regioisomeric cis/trans conjugated hydroxyeicosatetraenoic acids (HETEs), leukotrienes, lipoxins, and hepoxilins. Based on the predominant position of the incorporated of hydroperoxy group into arachidonic acid, lipoxygenases are classified as 5-, 8-, 12-, and 15-lipoxygenases (LOXs), with their respective main products as 5(S)-, 8(S)-, 12(S), and 15(S)-HETE. Arachidonic acid 5-lipoxygenation by 5-LOX is the rate-limiting step in the biosynthesis of leukotrienes and lipoxins, important mediators of many inflammatory processes. 15-LOX-2 uses arachidonic acid to form 15(S)-HETE [15] and a number of studies, including our own, have identified 15-LOX-2 suppresses tumor formation and growth by inducing tumor dormancy and cell cycle arrest [16].

## 2. Tumor Microenvironment in Carcinogenesis

Carcinogenesis is a multi-step, complex process leading to the development of a mass of malignant cells, or a tumor. By undergoing carcinogenesis, a tumor acquires the characteristics of most, if not all, of the hallmarks of cancer: sustained proliferative signaling, evasion of growth suppressors, replicative immortality, invasive ability and metastasis, induced angiogenesis, resistance to cell death, deregulation of cellular energetics, genomic instability and mutation, avoidance of immune destruction, and tumor-promoting inflammation [17]. In the past several decades, numerous studies have established the role of genetic alterations in a neoplasm’s acquisition of these major characteristics of cancer. The discovery of oncogenes and tumor suppressor genes, their respective signaling pathways, and mechanisms of pro-oncogenic genetic alterations, has provided direct explanations for the unregulated growth of a neoplasm and possible targets for anticancer therapies. Yet, despite knowing many of the critical genetic factors that drive a neoplasm’s development, many anticancer drugs for most forms of human cancers only provide transient relief of the disease [18]. This observation suggests that cancer genetics form only part of the whole picture of tumorigenesis and captures the motivation for further investigation into the surrounding stroma, which constitutes the tumor microenvironment (TME).

The TME is a highly dynamic, complex environment that evolves together with the multi-step tumorigenesis process. It is a joining of neoplastic cells, non-neoplastic cells including fibroblasts, immune cells, vascular endothelial cells, and non-cellular elements such as extracellular matrix (ECM) [19,20,21]. Individually, non-neoplastic components of the TME have multiple functions that may not appear as clearly pro-oncogenic as an oncogene. In fact, many of these functions are anti-oncogenic. However, during the process of multi-step tumorigenesis, these TME components provide functions that can collaborate with the oncogenic genetic changes. The TME may train a tumor into one of several possible molecular evolution pathways by signals originating in native and/or modified microenvironmental factors [22]. Traversing these collaborative pathways results in the neoplasm’s acquisition of the major characteristics of cancer. Simply put, tumorigenesis involves both alterations in gene expression and development of TME, as well as complex interactions between the two through complex and overlapping signaling pathways.

Herein we will describe the roles of prostaglandins, a class of bioactive lipids, in the formation and modulation of TMEs, with particular emphasis on their immune components, during tumorigenesis and the implications for cancer prevention and treatment. For prostaglandins in other aspects of cancer biology, there are several excellent reviews available [23].

## 3. Prostaglandin Regulation of Cancer-Associated Fibroblasts

One of the critical members of the TME is the fibroblast. A fibroblast is a mesenchymal cell mainly responsible for the maintenance and remodeling of the ECM, stimulation and regulation of inflammation, regulation of epithelial differentiation and proliferation, and wound repair [24,25]. In non-cancerous tissue, fibroblasts hold the tissues together and control their functions to maintain tissue homeostasis, especially after tissue damage [26]. Normally when tissue injury occurs, the damage triggers an inflammatory response. Many molecules involved in this response, such as growth factors and cellular adhesion molecules, trigger the activation of fibroblasts [27]. These activated fibroblasts, or myofibroblasts, produce ECM matrix components and matrix-modifying proteins, such as type I collagen and matrix metalloproteinases (MMPs), to remodel and repair the damaged tissue [27,28]. Additionally, these myofibroblasts secrete growth factors and cytokines themselves, such as hepatocyte growth factor (HGF), transforming growth factor-β (TGF-β), and interleukin-1 (IL-1), that modulate the inflammatory-immune response and the proliferation of epithelial cells [29,30]. The net effect of these secretions, in conjunction with the generation of contractile forces by the myofibroblast to close the wound, resolves the injury, which ends the inflammatory response, ceases fibroblast activation, and restores normal tissue function [28]. Thus, tissue homeostasis is maintained, preventing pathological conditions, such as infections and cancers, from developing.

However, in a setting with accumulated cellular stresses, such as chronic inflammation, the insult to the tissue is never fully resolved, leading to pathological, sustained activation of fibroblasts. In the context of cancer, the same fibroblasts that normally act to protect against tumorigenesis and invasion can be reprogrammed to promote tumorigenesis [21]. When a fibroblast undergoes such a reprogramming, it becomes known as a cancer-associated fibroblast (CAF). CAFs function in a similar manner to myofibroblasts in wound healing, secreting molecules to control and change the constitution of the tissue [20]. However, the net effect of the CAF’s actions creates a TME favorable for tumor growth, endowing a tumor with many of the major characteristics of cancer. Some pro-tumorigenic actions of CAFs include remodeling of the ECM, induction of angiogenesis, recruitment of inflammatory cells, secretion of immunosuppressive cytokines, secretion of growth factors, provision of metabolic support for cancer cells, and control of epithelial cell interactions with stroma [18,21,27,31]. Several studies have shown that activated fibroblasts can help in the initiation and promotion of tumors through modulating the TME [32,33,34].

It has been reported that COX2 expression in tumor epithelial cells was stimulated in response to inflammatory or stromal fibroblasts during the progression of ductal carcinoma in situ (DCIS) to invasive breast carcinomas in a tumor xenograft model [11]. In a co-culture model, inflammatory fibroblasts enhanced the motility and invasion of DCIS epithelial cells, with the NF-κB pathway identified as one of the mediators of stromal fibroblast-derived signals regulating COX2 expression in tumor epithelial cells. Inhibition of NF-κB and thus COX2 activity reduced the invasion-promoting effects of fibroblasts by ultimately downregulating the MMP-9. These findings support a role for COX2 produced by inflammatory or stromal fibroblasts in the TME in the progression of DCIS to invasive breast carcinomas [11].

In the TME of colon cancers, receptors of PGE_2_ (EPs), specifically EP_2_ and EP_4_, were identified as key targets for PGE_2_ to amplify inflammation and promote tumorigenesis. One study found that EP_2_ signaling elevates the expression of inflammation- and growth-related genes, such as TNFα, IL6, CXCL1, and Wnt5A. This elevation was significantly suppressed in EP_2_-deficient mice [35]. Other studies have demonstrated elevated EP_4_ levels in colorectal cancer, as well as tumor anchorage-independent growth [36] and drug resistance [37] via PGE_2_-EP_4_ signaling.

In another study, a population of COX2 expressing adventitial fibroblasts was found to remodel the lung immune microenvironment in the formation of the pre-metastatic niche [38]. The fibroblasts produced PGE_2_ to drive dysfunctional dendritic cells (DCs) and suppressive monocytes. The immune suppressive phenotypes of myeloid cells in the lung can be reversed through ablating of COX2 in the fibroblasts, which, similar to inhibiting the PGE_2_ receptors EP_2_ and EP_4_, can reduce lung metastasis in several breast cancer models [38]. This study suggests that fibroblasts can reprogram the microenvironment in the lung through a COX2/PGE2/EP_2_-EP_4_ pathway to facilitate cancer metastasis [38].

More studies are needed to define whether inhibition of the PGE_2_-EP_2_/EP_4_ signaling loop can be a valid approach to block tumorigenesis or treat pre-existing tumors, as well as whether EP_2_/EP_4_ play different functional roles in CAFs or infiltrating neutrophils.

## 4. Prostaglandin Regulation of Immune Constituents within the TME

### 4.1. Overview of Immune Phenotypes of the TME

The cells of the immune system are dynamic components of the TME. The immune cells are chiefly responsible for defense against foreign organisms and clearing away damaged tissue. Normally, these functions are tightly controlled by both feedforward and feedback control mechanisms to keep them in check, a process essential for tissue homeostasis. In TMEs, immune cells are important constituents of the tumor stroma and are active participants in the formation and evolution of TMEs during multi-step tumorigenesis.

Both innate and adaptive immune cells have been found in TMEs. Innate immune cells found in TMEs include macrophages, neutrophils, DCs, innate lymphoid cells, myeloid-derived suppressor cells (MDSCs), and natural killer (NK) cells, with most of them implicated in modulating tumor progression. Usually, the number of specific innate immune cells, such as M1-polarized macrophages and Batf3-dependent CD103+ sub-type DCs, is associated with favorable clinical outcomes [39,40]. In contrast, monocytes and M2-polarized macrophages within tumors promote the formation of an immunosuppressive environment and contribute to tumor growth, progression, and metastasis, leading to poor clinical outcomes [41].

The adaptive immune cells (T cells and B cells) within the TME can have huge impacts on tumor progression and response to treatments, particularly immunotherapies. Both cytotoxic T cells and helper T cells are found within tumor tissues. The cytotoxic T cells have been a target of immense interest due to their cytotoxic capabilities, which can be harnessed to kill tumor cells [42]. However, within tumor TMEs, those cytotoxic T cells are often anergic due to checkpoint controls. Current immunotherapies such as the inhibition of PD1-PDL1 immune checkpoints are intended to reactivate the anergic cytotoxic T cells to kill tumor cells [42,43].

In tumor tissues, several types of helper T cells, mainly Th1, Th2, Th9, Th17, and Th22 cells, have been found on the basis of their cytokine profiles. The differentiation of T cells is often referred to in a model called the “Th1/Th2 paradigm” [44], where Th1 cells drive a pro-inflammatory phenotype and Th2 cells contribute to tumor immune escape [45]. High levels of Th1 cells in the TME is associated with poor prognosis for patients with non-small cell lung cancer (NSCLC) [46]. Meta-analyses using The Cancer Genome Atlas (TCGA) data revealed that increased Th17 cells are generally associated with improved overall survival, but that Th1 cells are actually associated with worse OS across most immune subtypes of cancers [47]. Other helper T cells are implicated in tumor progression or in tumor responses to treatment, but more studies are needed to define their roles in TMEs.

Through immunogenomic analysis of over 10,000 tumors from 33 cancer types available from TCGA, one group identified six major subtypes of the immune landscape of cancer: Wound healing (C1), IFN-γ dominant (C2), inflammatory (C3), lymphocyte depleted (C4), immunologically quiet (C5), and TGF-β dominant (C6) [47]. The six immune subtypes are characterized by the differences in macrophage or lymphocyte signatures, Th1:Th2 cell ratio, extent of intra-tumoral heterogeneity, neoantigen load, aneuploidy, overall cell proliferation, expression of immunomodulatory genes, and prognosis [47].

It should be noted that the composition of immune cells within TMEs is regulated by the crosstalk between cancer cells, immune cells, and others, such as CAFs. Moreover, the composition of cells evolves during tumor growth and progression or in response to various treatments [44]. Prostaglandins, particularly PGE_2_, produced by tumor cells, as well as resident cells in the TMEs, can significantly impact the composition and function of immune cells within TMEs.

### 4.2. Prostaglandin Regulation of Immune Components in TMEs

#### 4.2.1. Prostaglandin E_2_

Known as a bioactive lipid for more than six decades, PGE_2_ generally promotes tumor growth and progression [12], particularly in gastrointestinal cancers [48,49]. Through its four cognate receptors, EP_1_, EP_2_, EP_3_, and EP_4_ [50] (Figure 1), PGE_2_ exerts multiple, often seemingly conflicting effects on different immune cells and many other cells [12,13]. While PGE_2_ supports local acute inflammation and phagocyte-mediated immunity, it can suppress both innate and antigen-specific immunity, such as the cytotoxic T lymphocytes and Th1- and NK cell-mediated immunity. PGE_2_ can suppress cytotoxic functions and IFN-γ production of NK cells by reducing IL-2, IL-12, and IL-15 activities [51,52]. In a rat model for lung metastasis by MADB106 syngeneic tumor cells, PGE_2_ suppressed NK activity in a dose-dependent manner and increased tumor cell retention in the lung [53]. High doses of PGE_2_ could increase lung metastasis fourfold, which can be abrogated by the selective depletion of NK cells [53]. Besides NK cells, the activities of other innate immunity components, such as macrophages, granulocytes, and mast cells, are also regulated by PGE_2_.

For the adaptive immune response, PGE_2_ can affect the functions of DCs, DC-T cell interaction, and T cell activation, depending upon the doses and engagement of the different EP receptors. PGE_2_ can suppress the differentiation of Th1-inducting DCs at an early stage, leading to their dysfunctions in cancer [54,55]. PGE_2_ can further drive them to myeloid-derived suppressor cells [56], which in turn suppress the functions of cytotoxic T cells. Interestingly, PGE_2_ has stimulatory effects on fully developed DCs cells in their homing to lymph nodes and priming of naive T cells. But the DCs matured by PGE_2_ preferentially produced Th2 responses and exhibited impaired capacity to induce the CTL- or NK cell-mediated type 1 immunity [57].

PGE_2_ inhibits the production of Th1 cytokines but not those associated with Th2 [58]. PGE_2_ can selectively reduce the levels of IFN-γ, a Th1 cytokine, while having minimal effects on the productions of IL4 and IL5, two Th2 cytokines, in CD4^+^ T helper cells [58,59]. Further, PGE_2_ can also dampen Th1 responses by reducing the production of or responsiveness to IL-12 [60,61], a cytokine essential in the induction of the Th1 response and reversal of the Th2 response [62]. Therefore, increased PGE_2_ can dampen the Th1 response and tilt the balance toward the Th2 responses or other forms of immune response.

Among the four cognate receptors for PGE_2_, EP_2_ and EP_4_ signaling cause immunosuppression through the recruitment and activation of regulatory T (Treg) cells, while concurrently promoting local inflammation through activating NF-kB in myeloid cells [63]. In general, PGE_2_ promotes acute local inflammatory responses and phagocyte-mediated immunity in response to the presence of pathogens. However, PGE_2_, especially at elevated doses, suppresses the cytotoxic immune responses of CTL, Th1, and NK cells. Therefore, enhanced PGE_2_ levels in tumor tissues can lead to an immunosuppressive TME.

#### 4.2.2. Prostaglandin I_2_

The role of PGI_2_ in modulating inflammation and the immune response has been indicated by the increased inflammation in mice with deficiency in the PGI_2_ receptor (IP). Mice with deficient IP exhibited increased severity, with increased production of the Th1 cytokine IFN-γ in the lung after a viral infection [64]. In common with PGE_2_, PGI_2_ and its analogs can inhibit the proliferation and activation of T and B lymphocytes. Dentritic cells treated with iloprost, a PGI_2_ analog, promote Treg differentiation in mice and suppress the DC-mediated airway inflammation [65]. In an in silico analysis using multiple datasets from Oncomine, it was found that the expression of PGI_2_ synthase (PTGIS) was associated with the infiltration of tumor-associated macrophages and Treg cells [66]. However, more studies are needed to determine its precise role in overall tumor progression, as well as its particular role in modulating immune responses and the TME.

#### 4.2.3. Prostaglandin D_2_

Converted from PGH_2_ by PGD synthase (PGDS), PGD_2_ is pro-inflammatory and implicated in allergic disease. Through its cognate receptors DP1 and DP2 (also known as CRTH2), PGD_2_ can modulate cytokine production in DCs. With DP2 preferentially expressed in Th2 lymphocytes and other immune cells, PGD_2_ can regulate chemotaxis and type 2 cytokine production in the inflammatory response [67,68].

#### 4.2.4. Thromboxane A_2_

A strong activator of platelets, TXA_2_ exerts its biological activities through its cognate receptor TP. TXA_2_ is produced by thromboxane A_2_ synthase (TBXAS1) using the substrate PGH_2_. Activated platelets, DCs, and macrophages are major cellular sources of TXA_2_. Our previous study found that prostate cancer cells express thromboxane A_2_ synthase and produce TXA_2_ to modulate tumor cell motility [10]. Inhibitors of both COX1 and COX2 are needed to abolish TXA_2_ production in prostate tumor cells [10]. One study found that TXA_2_ negatively regulated interactions between DCs and T cells and modulated acquired immunity [69]. Further, enhanced immune antigen responses were observed in TP-deficient mice [69]. However, further studies are needed to determine whether TXA_2_ plays a role in the formation or modulation of immunosuppressive TMEs.

#### 4.2.5. Prostaglandin F_2α_

PGF_2α_ is produced by PGF_2α_ synthase (PGFS) utilizing PGH_2_ as the substrate. Its cognate receptor, the FP receptor, can also bind other prostaglandins in addition to PGF_2α_ [70]. PGF_2α_-FP plays important roles in many physiological and pathological situations, such as ovulation, parturition [71], renal function, myocardial dysfunction, and pain [72]. FP agonists are used to reduce the intraocular pressure of glaucoma [73]. Injection of PGF_2α_ into animals can cause acute inflammation, which is correlated with the formation of reactive radicals such as isoprostanes [74]. In endometrial adenocarcinoma, PGF_2α_-FP receptor signaling promotes neutrophil chemotaxis via regulating CXCL1 [75]. Interestingly, in an HCl-induced mouse model for acute lung injury and respiratory distress syndrome, the inhibition of FP receptors increased neutrophil migration into the lungs, leading to increased lung inflammation [76]. Further studies are needed to determine the role of PGF_2α_-FP in the tumor inflammatory microenvironment.

## 5. Role of Cyclooxygenases and Prostaglandins in Tumor Evasion of Immune Surveillance

Evasion of immune surveillance is one of the hallmarks of cancers [17]. There are many mechanisms for tumors to escape immune surveillance, including, but not limited to, upregulation of CD4^+^ Treg cells, MDSCs, M2 macrophages, immunosuppressive mediators, as well as immune editing, tolerance, and deviation [77]. For example, the Treg cells can suppress cytotoxic effector cells, NK cells, and DCs to restore immune homeostasis after inflammation. In tumors, Treg cells can be recruited by tumor cells or tumor-associated macrophages (TAM, mainly M2 macrophages) and become part of the TME to mold its response to immune surveillance. The suppression of the T cell-mediated immune responses by Treg cells can be achieved through the secretion of immunosuppressive cytokines such as IL-10, IL-35 and TGF-β. Another pathway to suppress effector T cells by Tregs is through metabolic disruption, such as the sequestration of IL-2 by IL-2α/CD25 on the surface of Tregs, leading to the apoptosis of T cells.

In a seminal study, it was demonstrated that cyclooxygenases play an important role in tumor evasion of immune surveillance by producing prostaglandins, particularly PGE_2_ [14]. First, they found that the conditioned media from Braf(V600E) mouse melanoma cells have immunomodulatory effects on myeloid cells and then identified prostaglandins, particularly PGE_2_, as the major immunomodulatory factor from the Braf(V600E) mouse melanoma tumor on myeloid cells [14]. Next, through the genetic ablation of cyclooxygenases (COX) in Braf(V600E) mouse melanoma cells or in N-Ras(G12D) melanoma or in breast or colorectal cancer cells, they demonstrated that those cells, with COX expression deleted and hence the biosynthesis of prostaglandins abolished, could not grow well in the immune-competent mice but that they grow equally well in the immunocompromised mice [14]. Among the prostaglandins involved, the PGE_2_ pathway was demonstrated as the facilitator of immune evasion because genetic ablation of PGE synthase rendered Braf(V600E) mouse melanoma cells susceptible to immune controls in a manner similar to COX ablation [14]. These findings suggest that COX and prostaglandins, particularly PGE_2_, help tumors to escape from immune surveillance.

The engagement of programmed cell death protein 1 (PD-1) by its ligand PD-L1 plays a major role in the anergy of activated T cells during tumor escape from immune surveillance. The expression of PD-1 and PD-L1 can be influenced by many factors [78], including tumor-associated macrophages [79] and multiple cytokines with different signaling pathways involved [80]. While PD-L1 can be expressed by tumor cells, one group found an increased expression of PD-L1 in murine bone marrow cells when cocultured with bladder cancer cells [81]. Tumor-induced PD-L1 expression was found in F4/80+ macrophages and Ly-6C+ myeloid-derived suppressor cells [81]. Tumor infiltrating PD-L1 expressing cells isolated from tumor bearing mice had high expression levels of COX2 and microsomal PGE_2_ synthase 1, and inhibition of COX2/mPGES1/PGE_2_ pathway reduced PD-L1 expression [81]. The studies suggest that this PGE_2_ pathway is involved in the regulation of PD-L1 expression in tumor infiltrating myeloid cells, contributing to immune suppressive TME and tumor evasion of immune surveillance.

A recent study confirmed the role of the COX2/PGE_2_/EP_4_ signaling loop in tumor evasion of immune surveillance in colorectal adenoma [82]. In this study, exogenously added PGE_2_ stimulated PD1 expression in mouse splenic cytotoxic T cells and THP-1 derived macrophages [82]. Inhibition of COX2 with Celecoxib or EP_4_ with Ono-AE3–208 in APC^min/+^ mice reduced the PD1 expression in intestinal macrophages and cytotoxic T cells, and stimulated cytotoxic T cell activation and macrophage phagocytosis [82].

With the availability of TCGA cancer genomics databases, together with RNAseq data, we examined the potential association of COX-prostaglandin signaling with gene signatures that might have an impact on the immune components of TME, such as PD-L1 (CD274). As shown in Table 1, in human lung adenocarcinoma (TCGA, PanCancer Atlas, 510 patients/samples), CD274 mRNA levels are positively correlated with COX1 (PTGS1), EP2 (PTGER2), EP4 (PTGER4), and DP (PTGDR) with Spearman’s or Pearson’s coefficients over 0.3 and the coefficient of determination (R^2^) over 0.1. Interestingly, EP2 and EP4 levels were found to be correlated with PD-1 levels in infiltrating CD8+ T cells in lung cancer [83].

In contrast to PGE_2_/EP2/EP4, other prostaglandins and their receptors have not been studied in detail for their potential roles in tumor immune evasion.

## 6. Translational Potential and Perspective

Prostaglandins have complex and sometimes paradoxical effects on inflammation and immune responses. The same prostanoid formed by COX1 or COX2 may promote or suppress inflammation, depending upon their different spatial and temporal contexts. Several studies suggest that the COX2-PGE_2_ pathway contributes to the formation of immunosuppressive TMEs. While more studies are needed to delineate the precise roles of prostaglandins in the TME of different cancers, the biggest question is whether we can overcome or reverse the immune suppression of TMEs and enhance the efficacy of immunotherapy through targeting prostaglandins and their signaling effectors. Indeed, inhibition of COX2 or EP4 can lead to the restoration of NK functions to reduce the metastatic burden of breast cancers [84], inhibit M2 macrophage differentiation, enhance CTL-mediated cytotoxicity, and drive TME to favor the Th1 immune responses [85,86].

It should be noted that NSAIDs, including aspirin and COX2 specific inhibitors, are among the most consumed drugs. Further, there are many analogues of PGE_2_, PGF_2α_ and PGI_2_, TP antagonists, as well as antagonists of DP1, DP2 and EP4, undergoing clinical evaluations for various indications. More studies are needed to determine whether and how these drugs can be repurposed to reduce tumor evasion of immune surveillance and to enhance the efficacy of immunotherapy of various cancers.

## Figures and Tables

**Table 1:** Association of CD274 (PD-L1) expression with COX-prostaglandin signaling pathway in human lung adenocarcinoma (TCGA, PanCancer Atlas, 510 patients/samples).

	Gene Name	R^2^ with CD274 (Log Scale)	Spearman Coefficient	Pearson Coefficient
COX1	PTGS1	0.17	0.43, *p* = 8.41 × 10^−24^	0.41, *p* = 1.75 × 10^−22^
COX2	PTGS2	0	−0.04, *p* = 0.313	−0.02, *p* = 0.679
PGE_2_ pathway				
mPGES1	PTGES	0	0, *p* = 0.966	−0.01, *p* = 0.741
PTGES2	PTGES2	0.01	−0.13, *p* = 3.79 × 10^−3^	−0.11, *p* = 0.0112
PTGES3	PTGES3	0	0.02, *p* = 0.578	0.04, *p* = 0.423
EP1	PTGER1	0	0.01, *p* = 0.842	0.01, *p* = 0.755
EP2	PTGER2	0.1	0.34, *p* = 2.80 × 10^−15^	0.31, *p* = 7.61 × 10^−13^
EP3	PTGER3	0	−0.00, *p* = 0.977	−0.03, *p* = 0.467
EP4	PTGER4	0.22	0.49, *p* = 1.83 × 10^−32^	0.47, *p* = 9.52 × 10^−29^
PGD_2_ pathway				
PTGDS	PTGDS	0.03	0.21, *p* = 1.053 × 10^−6^	0.19, *p* = 2.551 × 10^−5^
DP	PTGDR	0.11	0.37, *p* = 9.28 × 10^−18^	0.33, *p* = 7.73 × 10^−15^
DP2	PTGDR2	0.01	−0.10, *p* = 0.0261	−0.12, *p* = 6.516 × 10^−3^
TXA_2_ pathway				
TBXAS1	TBXAS1	0.05	0.23, *p* = 2.20 × 10^−7^	0.23, *p* = 2.44 × 10^−7^
TP	TBXA2R	0.04	0.17, *p* = 1.070 × 10^−4^	0.19, *p* = 1.580 × 10^−5^
PGI_2_ pathway				
PGI_2_ synthase	PTGIS	0.02	0.19, *p* = 1.220 × 10^−5^	0.15, *p* = 9.574 × 10^−4^
IP	PTGIR	0.03	0.19, *p* = 2.412 × 10^−5^	0.16, *p* = 3.604 × 10^−4^
PGF_2a_ pathway				
PGF synthase (predicted)	PRXL2B	0	−0.05, *p* = 0.216	−0.04, *p* = 0.419
FP	PTGFR	0.03	0.20, *p* = 4.658 × 10^−6^	0.17, *p* = 9.216 × 10^−5^

## Data Availability

Not applicable.
